# Cutting-Edge Smart Hydrogel Platforms for Improved Wound Healing

**DOI:** 10.3390/pharmaceutics18040406

**Published:** 2026-03-25

**Authors:** Ameya Sharma, Vivek Puri, Divya Dheer, Malkiet Kaur, Kampanart Huanbutta, Tanikan Sangnim

**Affiliations:** 1 Chitkara University School of Pharmacy, Chitkara University, Baddi 174103, India; ameya.sharma@chitkarauniversity.edu.in (A.S.); vivek.puri@chitkarauniversity.edu.in (V.P.); divya.dheer@chitkarauniversity.edu.in (D.D.); 2 Maharishi Markandeshwar College of Pharmacy, Mullana University, Mullana 133207, India; malkietkaur033@gmail.com; 3 Department of Manufacturing Pharmacy, College of Pharmacy, Rangsit University, Pathum Thani 12000, Thailand; kampanart.h@rsu.ac.th; 4 Faculty of Pharmaceutical Sciences, Burapha University, Chonburi 20131, Thailand

**Keywords:** smart hydrogel, wound healing, drug delivery system, tissue engineering, nanomaterials

## Abstract

**Background/Objectives:** Wound management presents a substantial clinical challenge due to the rising incidence of chronic wounds, infections, and the limitations of conventional dressings in creating an ideal healing microenvironment. This review aims to provide a comprehensive overview of advanced smart hydrogel platforms designed to improve wound healing outcomes, focusing on their capacity to respond adaptively to physiological and external stimuli. **Methods:** This article analyzes the core characteristics of smart hydrogels, specifically examining stimuli-responsive systems (pH, temperature, enzyme, light, and electricity). The review evaluates advanced configurations—including injectable, self-healing, and 3D-printable systems—and functionalized hydrogels integrated with antimicrobials, drugs, and nanocomposites. Additionally, essential characterization methodologies, biological assessments, and regulatory considerations for clinical translation are synthesized. **Results:** The literature, which is predominantly preclinical in nature, indicates that functionalized hydrogels significantly enhance tissue regeneration, angiogenesis, and infection control compared to traditional methods. Conductive hydrogels utilizing bioelectrical signals show particular promise in accelerating the healing process. While current clinical applications and commercial products demonstrate efficacy, significant barriers remain regarding large-scale manufacturing and regulatory approval. **Conclusions:** Smart hydrogels represent a transformative approach to precision wound management, offering superior adaptability and therapeutic delivery. To achieve widespread clinical adoption, future research must address manufacturing scalability and focus on emerging trends, such as the integration of biosensors and AI-powered monitoring systems, to create fully intelligent wound care solutions.

## 1. Introduction

Restoring tissue integrity during wound healing is a dynamic biological process that requires an intricate system of interconnected biochemical, molecular, and cellular events [1]. Despite substantial advances in biomedical science, the management of both acute and chronic wounds remains a major clinical and economic challenge on a global scale. Infection, necrosis, and poor tissue regeneration are common complications of surgical sites, pressure injuries, diabetic complications, peripheral vascular disease, and traumatic wounds [2]. Traditional wound dressings such as gauze and polymeric films mainly provide passive protection and moisture retention but lack the ability to actively regulate the wound microenvironment [3]. In certain instances, the wound milieu may be negatively impacted by inadequate moisture balance, limited antimicrobial efficacy, or insufficient exudate management. Although advanced wound therapies are intended to actively regulate the wound microenvironment, their composition, responsiveness, and clinical application can result in both beneficial and unintended effects [4]. As a result, the need for advanced wound care systems that can overcome the drawbacks of conventional treatments and actively contribute to the healing process is on the rise [5,6,7]. Chronic wounds are often subsequent indications of underlying systemic illnesses rather than isolated local tissue abnormalities. Conditions include diabetes mellitus, peripheral artery disease, chronic venous insufficiency, autoimmune illnesses, and extended pressure exposure significantly disrupt perfusion, immunological response, metabolic regulation, and cellular repair processes [8]. Therefore, local treatment alone cannot accomplish adequate wound healing. The underlying disease process must be treated concurrently for effective management, including vascular restoration, infection control, pressure redistribution, glycemic control, and systemic inflammation reduction. Advanced wound dressings, such as smart hydrogels, are to be regarded as supplementary procedures within a holistic, multidisciplinary therapy framework rather than as independent curative remedies [9,10].

The complex nature of wounds and the many factors that can affect their healing have led to the development of innovative wound coverings. Prolonged inflammation is a common symptom of chronic wounds caused by factors such as microbial burden, oxidative stress, hypoxia, and limited angiogenesis [11]. Topical and systemic antimicrobial treatments, particularly antibiotics, are significantly limited in their effectiveness due to the emergence of drug-resistant bacterial strains and the formation of biofilms [12]. In contrast, for commonly used antiseptics such as hypochlorite solutions, clinically relevant bacterial resistance at concentrations applied in routine practice has not been conclusively demonstrated, although experimental studies have explored potential adaptive responses under laboratory conditions [13]. Maceration of neighboring tissues can occur in wounds caused by an excess of exudate, but cellular migration and granulation are both impeded in dry wounds due to a lack of moisture [14]. The ability to adhere to uneven anatomical surfaces, allow gaseous exchange, maintain proper moisture balance, and avoid unpleasant dressing changes is also essential for many patients. The importance of multifunctional materials that can promote regulated, supportive, and physiologically interacting wound healing is further highlighted by these requirements [15].

One of the most promising groups of biomaterials for advanced wound care is smart and functionalized hydrogels, which have developed in response to these requirements. Hydrogels are networks of three-dimensional hydrophilic polymers that can absorb and hold large volumes of water without deteriorating structurally [16]. They provide an ideal setting for cell migration, proliferation, and tissue regeneration due to their biomimetic qualities, high moisture content, and similarity to the original extracellular matrix (ECM) [17]. In addition to their inherent structural advantages, smart hydrogels have stimuli-responsive properties that allow them to actively interact with the wound environment. In response to internal or external triggers, such as pH, enzymes, reactive oxygen species, glucose, or temperature, these hydrogels can go through physicochemical changes like swelling, breakdown, gel–sol transition, or controlled drug release. This level of responsiveness enhances the precision and efficacy of wound therapy by enabling real-time modification to the wound’s pathological condition [3,18].

Hydrogels are made even more useful through functionalization, which involves adding bioactive compounds, nanoparticles, antimicrobial agents, growth factors, peptides, extracts from plants, and gene delivery systems to the polymer matrix [19]. Hydrogels shift from being inert support structures to dynamic therapeutic platforms due to their multifunctionality. A decrease in systemic adverse effects and an increase in local bioavailability can be made possible with the controlled and sustained release of therapeutic drugs directly at the wound site through functionalized hydrogels. They have the ability to reduce inflammation, stimulate blood vessel formation, prevent the spread of bacteria, neutralize excess free radicals, and aid in the re-epithelialization and collagen deposition processes [20]. The vast array of synthetic and natural polymers available in polymer chemistry allows for remarkable control over mechanical strength, degradation rate, porosity, adhesiveness, self-healing capabilities, and injectability. Some examples of these polymers are hyaluronic acid, chitosan, alginate, gelatin, and poloxamers. Hydrogels are able to respond to a wide range of clinical limitations due to their tunable physicochemical properties. These include deep wounds, burns, diabetic ulcers, and post-surgical defects [21].

Patient comfort and better healing results are enhanced by the benefits of smart hydrogels in moisture control, oxygen permeability, biocompatibility, and reduced dressing change frequency [22]. The cellular processes essential for wound closure are expedited by their ability to generate a controlled moist wound environment, which also accelerates autolytic debridement. However, it is crucial to acknowledge that not all chronic lesions experience uniform benefits from sustained moisture [23]. Excessive moisture may contribute to bacterial proliferation, maceration, or compromised tissue perfusion in specific clinical conditions, such as lesions infected with Pseudomonas aeruginosa or those associated with peripheral arterial disease (PAD) [24]. Clinically, it may be advantageous to implement meticulous exudate management and a wound environment that is relatively drier in such instances. Consequently, it is imperative to select and customize sophisticated hydrogel systems in accordance with the distinctive pathophysiological condition and wound etiology [10]. Smart hydrogels enhance wound treatment by regulating moisture, ensuring biocompatibility, and enabling tailored delivery of medicinal medicines. Their therapeutic efficacy is significantly influenced by their composition, crosslinking density, degradation kinetics, and the particular wound pathophysiology [19,25]. The biological response to wound hydration is contingent upon the specific cell type involved. Fibroblasts typically necessitate an adequately moist milieu to facilitate motility, extracellular matrix deposition, and granulation tissue development. Conversely, keratinocyte proliferation and differentiation during re-epithelialization are enhanced under physiologically balanced settings, characterized by moderate hydration levels (about 60–70%) rather than excessive moisture. Sustained overhydration may hinder the repair of the epithelial barrier. Thus, smart hydrogels should not be seen as generally advantageous; instead, their design must be customized to the wound stage, microbial load, vascular condition, and cellular needs to attain best therapeutic results [10,26]. Furthermore, the transparency of certain hydrogel formulations enables non-invasive visual monitoring of the wound without removing the dressing [27]. By improving their adhesiveness and ability to heal on their own, hydrogels have become more useful in the clinic; these innovations allow for prolonged stability, even when subjected to mechanical stress, and ensure direct contact with wound surfaces. Also, 3D-printed hydrogel structures, hydrogel-based tissue engineering scaffolds, and hybrid systems combining nanoparticles, sensors, and drug delivery modules have all been made possible by recent bioengineering advancements. Personalized and precision-based wound care strategies are becoming more popular, and these advances are just a part of it [28,29].

Innovative approaches for developing smart dressings that can monitor the condition of wounds in real time have emerged primarily through the combination of hydrogel design with nanotechnology, bioprinting, and stimuli-responsive chemistry [30]. As an example, hydrogels that contain chromophores that respond to changes in pH can be used as markers to identify infections. Electrical stimulation therapy, made possible by conductive hydrogels, can speed up tissue regeneration by increasing angiogenesis [31]. With the use of thermo-responsive hydrogels, small changes in temperature can trigger the release of drugs whenever the patient needs them. Hydrogels filled with antioxidants have a similar effect, neutralizing reactive oxygen species that hurt chronic wounds and prevent them from healing. All of these new innovations show how smart hydrogels can solve so many problems in wound care that have been prevalent for many years [32,33].

The variability of polymer properties, difficulties in scaling up and manufacturing, regulatory concerns, long-term safety, and the necessity for thorough clinical validation are some of the challenges that smart and functionalized hydrogels continue to confront as they make their way from the lab to the clinic, despite considerable progress [34]. Nevertheless, new hydrogel-based wound dressings that are more effective, stable, and economical are anticipated to be developed as a result of the ongoing integration of materials science, biomedical engineering, pharmaceutics, and regenerative medicine [35]. In this review article, we comprehensively discuss the classification, design strategies, functionalization approaches, therapeutic mechanisms, recent advancements, clinical application and future perspectives of smart and functionalized hydrogels for wound healing.

## 2. Overview of Smart Hydrogels

Smart hydrogels, also known as stimulus-responsive or intelligent hydrogels, are a sophisticated class of soft biomaterials that may alter physicochemical properties in predictable and reversible ways in response to specific environmental stimuli. In contrast to conventional hydrogels, which maintain a fixed structure post-formation, smart hydrogels can detect environmental changes and convert these modifications into functional responses, including swelling, contraction, degradation, self-healing, sol–gel transitions, or the controlled release of embedded therapeutic agents [36]. Smart hydrogels are incredibly versatile and have the potential to revolutionize various fields of biomedical engineering. They have the power to heal wounds, regenerate tissues, distribute drugs, sense biosignatures, and even provide individualized medical care. Mechanical strength, degradation kinetics, porosity, and biological activity can be precisely controlled due to their programmability, customizable network architecture, and compatibility with a wide range of natural and synthetic polymers [37].

### 2.1. Definition and Key Features of Smart Hydrogels

In general, smart hydrogels are three-dimensional crosslinked polymeric networks that modify their physicochemical properties in response to internal or external stimuli. The hydrophilic functional groups found in these polymers, including hydroxyl, amide, carboxyl, and sulfate, allow them to absorb a lot of water, producing structures that are similar to the extracellular matrix (ECM) in texture and appearance [38]. The “smart” feature develops when functional moieties, dynamic covalent bonds, reversible crosslinkers, or responsive polymer chains that can undergo conformational changes in response to certain stimuli are incorporated [39].

Smart hydrogels differ from regular hydrogels in several important ways. One is that they can respond to physical, chemical, or biological stimuli in a way that allows them to undergo reversible structural changes like swelling, de-swelling, degradation, network cleavage, or controlled release of encapsulated agents [40]. In addition to being programmable and reversible, these materials can go back to their original condition when stimuli are removed. The responsiveness can be fine-tuned by adjusting the polymer composition, crosslinking density, and functional group structure. Their biomimicry and biocompatibility make them highly suitable for use in biomedicine; they can be made from either natural or synthetic polymers, and their high water content, softness, and ECM-like structure make them mimic the properties of native tissue [41]. By adding nanoparticles, nanofibers, or dynamic covalent interactions, smart hydrogels can have their strength and elasticity adjusted to withstand physiological demands. This makes them very mechanically tunable. By modulating porosity, permeability, and degradation based on responses, they may release therapeutic drugs site-specifically and sustainably with minimally invasive methods [42]. This reduces systemic toxicity and increases the effectiveness of treatments. The capacity to self-repair structural defects and sustain functional stability over extended periods of use is conferred to some formulations by reversible dynamic linkages such as disulfide bonds, Schiff base reactions, and host–guest interactions. For next-gen wound dressings, smart hydrogels are a great platform due to their multifunctionality and ability to be tailored to the specific needs of the wound microenvironment [43].

### 2.2. Mechanisms of Responsiveness in Smart Hydrogels

The responsiveness of smart hydrogels originates from the chemical functionalities and molecular architecture of their polymer networks. External or internal stimuli can induce physicochemical changes such as ionization, polymer chain collapse, reversible bond dissociation, or crosslink cleavage, ultimately altering the hydrogel structure and behavior [44].

Smart hydrogels can respond to a wide range of environmental cues, enabling their application in wound healing, drug delivery, and tissue engineering. Among these systems, pH-responsive hydrogels contain ionizable functional groups that alter network swelling and drug release in response to pH changes commonly associated with infection or inflammation [44,45,46,47]. Temperature-sensitive hydrogels, such as those based on poly(N-isopropylacrylamide) (PNIPAM), exhibit a lower critical solution temperature (LCST), enabling reversible sol–gel transitions and controlled therapeutic release in response to localized thermal variations [48,49]. Enzyme-responsive hydrogels incorporate degradable linkages that can be cleaved by enzymes such as matrix metalloproteinases (MMPs), which are frequently overexpressed in chronic wounds, thereby facilitating targeted drug release and tissue regeneration [50,51,52].

In addition, oxidative stress-responsive hydrogels react to elevated levels of reactive oxygen species (ROS), enabling the delivery of antioxidants or therapeutic agents within oxidative microenvironments associated with tissue damage and inflammation [53,54]. Glucose-responsive hydrogels can detect glucose concentrations through enzymatic or receptor-based mechanisms and have been explored for applications such as insulin delivery in diabetic wounds [55]. Furthermore, electrically, magnetically, and light-responsive hydrogels enable external regulation of swelling behavior, mechanical properties, or drug release through applied physical stimuli, providing additional control over therapeutic interventions [56,57,58]. To further enhance adaptability, multi-stimuli-responsive hydrogels combine two or more responsiveness mechanisms—such as pH and temperature or glucose and oxidative stress—allowing these systems to dynamically adjust to complex physiological conditions and better mimic the natural wound microenvironment [6,59].

## 3. Types of Smart Hydrogels for Wound Healing

Smart hydrogels represent a novel class of biomaterials engineered to actively interact with the wound environment and respond to internal or external stimuli, thereby improving therapeutic outcomes [60]. Unlike conventional passive wound dressings, smart hydrogels exhibit adaptive functionalities that enable them to detect physicochemical variations, deliver therapeutics in a regulated manner, modify their structure, and interact with biological processes that facilitate natural healing [61]. These materials integrate principles of polymer chemistry, biotechnology, molecular engineering, and responsive material design to enable advanced wound management capabilities, including sustained moisture retention, infection prevention, promotion of angiogenesis, cellular infiltration, and controlled degradation [62]. As wound biology is complex and dynamically influenced by infection, inflammation, tissue degradation, and regenerative processes, smart hydrogels are designed to provide stimulus-responsive treatments tailored to specific healing stages and conditions [63].

Despite these promising characteristics, the practical application of smart hydrogels remains challenging. The feasibility of combining specific hydrogel matrices with therapeutic agents is often constrained by chemical and physical incompatibilities, and it remains uncertain which types of smart hydrogels can be effectively integrated with particular active ingredients [64]. Interactions between the hydrogel network and incorporated bioactive agents may compromise the stability, responsiveness, or biocompatibility of the hydrogel matrix. In addition, certain sterilization techniques may reduce the potency of incorporated therapeutics or alter the functional properties of the smart hydrogel, including its stimulus-responsive behavior [65]. Consequently, careful selection of hydrogel materials and therapeutic agents, together with appropriate processing and sterilization strategies, is essential to ensure the safety, functionality, and therapeutic efficacy of smart hydrogel systems in wound healing applications [66].

**Figure 1:**
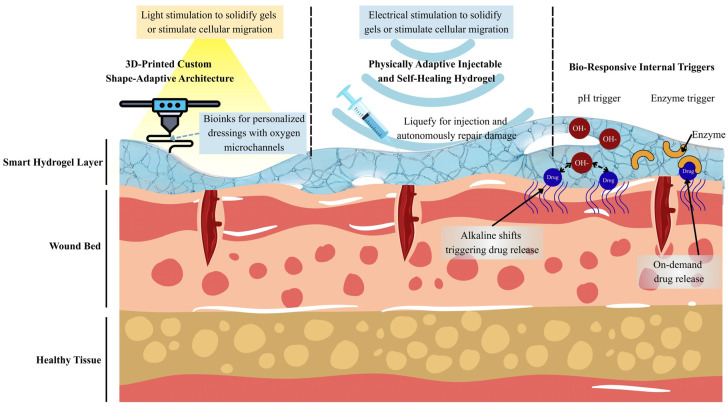
Design strategies of smart hydrogels for wound healing, including stimuli-responsive, injectable self-healing, and 3D-printed personalized dressings.

To address the diverse challenges of dynamic wound healing, various advanced design strategies have been explored to develop next-generation smart hydrogel platforms. These strategies aim to enhance responsiveness, adaptability, and therapeutic performance in complex wound environments. As illustrated in Figure 1, the primary approaches discussed in this section include stimuli-responsive hydrogels, injectable self-healing hydrogels, and 3D-printed personalized dressings.

### 3.1. Stimuli-Responsive Hydrogels

Stimuli-responsive hydrogels, often termed “intelligent” or “smart” hydrogels, exhibit the capacity to alter their physical characteristics, swelling behavior, internal porosity, degradation profile, or drug release kinetics in response to defined stimuli [67]. These triggers may stem from the wound itself, including alterations in pH, temperature, and enzyme activity, or may be externally administered through the use of light or electrical energy [68]. Given that wound environments are dynamic and frequently fluctuate due to microbial activity, inflammation, as well as therapeutic interventions, stimuli-responsive hydrogels function as real-time modulators that facilitate more precise, site-specific, and temporally controlled therapeutic delivery [69]. This capability is especially crucial for chronic lesions, which often necessitate frequent modifications in antimicrobial concentration, growth factor levels, and degradation rate to correspond with the healing process [70]. The primary categories of stimuli-responsive hydrogels pertinent to wound management include pH-sensitive, thermoresponsive, enzyme-reactive, light-triggered, and electrically responsive systems, which are engineered to capitalize on specific physiological signals or externally applied stimuli [71].

#### 3.1.1. pH-Responsive Hydrogels

Among the numerous biological variables influenced during the wound healing process, pH stands out as one of the most valuable indicators of the condition of tissues [72]. Normal skin sustains a slightly acidic pH; however, chronic wounds, infected ulcers, and severe inflammatory lesions often demonstrate a shift toward alkaline conditions due to bacterial proliferation, tissue breakdown, and metabolic by-products [73]. pH-responsive hydrogels leverage these predictable variations to modulate their behavior at the lesion site. They are generally composed of polymers bearing acidic or basic functional groups that can undergo ionization in response to ambient pH [74]. When subjected to pH levels outside the typical range of skin, these polymers experience variations in charge density that modify their swelling behavior, osmotic pressure, as well as polymer chain mobility [75]. Elevated ionization levels typically result in enhanced electrostatic repulsion among polymer chains, prompting the hydrogel to uptake water and undergo expansion [76]. This enlargement may induce accelerated drug release, enhance wound cushioning, augment exudate absorption, and improve conformity to irregular surfaces. In chronic or infected lesions characterized by elevated pH levels associated with heightened microbial activity, such hydrogels are capable of releasing antimicrobial agents, metal ions, or anti-inflammatory compounds in proportion to the extent of the dysfunction [77,78]. Their capacity to respond to biochemical conditions renders them valuable not only for therapeutic delivery but also for real-time sensing and feedback systems in wound management [79].

As summarized in Table 1, numerous pH-responsive hydrogel systems have demonstrated promising therapeutic potential in wound healing. For example, injectable double-network hydrogels enabling pH-responsive resveratrol release have shown antibacterial and antioxidant effects while enhancing angiogenesis and re-epithelialization in chronic wound models [80]. Other multifunctional systems, including H-CuS@EGCG hydrogels and CMCS/2-FPBA/TA-Fe hydrogels, have exhibited strong antibacterial activity, anti-inflammatory effects, and improved tissue regeneration in infected or diabetic wound models [81,82]. Additional platforms incorporating nanozymes, enzyme-responsive networks, or pH-responsive drug delivery systems have further demonstrated enhanced angiogenesis, immunomodulation, and accelerated wound closure [83,84,85,86].

#### 3.1.2. Temperature-Responsive Hydrogels

Temperature fluctuations within a laceration indicate significant physiological changes, such as infection, inflammation, enhanced blood circulation, or fever [67]. Temperature-responsive hydrogels leverage these fluctuations or the stable temperature of the human body to facilitate sol–gel transitions, thereby allowing minimally invasive application [87]. Numerous thermosensitive hydrogels are engineered to maintain a liquid or semi-liquid state at ambient temperature, facilitating their administration through injection or topical application. Upon attaining body temperature, they promptly shift into a more solid or gelled state as a result of alterations in hydration levels and polymer chain interactions [88]. This phenomenon of lower critical solution temperature appears frequently in polymers including poly(N-isopropylacrylamide), Pluronic-based copolymers, as well as modified polysaccharides. When applied to a laceration, these hydrogels adapt precisely to the surface of the lesion, fill cavities, and establish a stable protective barrier without the need for external curing procedures [89]. Their capacity to function as injectable depots assures that therapeutic agents comprising stem cells, growth-promoting enzymes, or antibiotics are retained locally and are not lost through discharge or dressing displacement [90]. The gel structure formed by thermal induction also offers a moist healing environment and safeguards against external contaminants. Because numerous infected lesions produce localized warmth, temperature-responsive hydrogels can also be designed to enhance drug release under these conditions, thereby offering increased therapeutic efficacy when the risk of infection is elevated [91]. Their intuitive administration, minimal invasiveness, and physiological compatibility have contributed to their widespread adoption in regenerative medicine applications [92].

Several thermoresponsive hydrogel systems have shown promising therapeutic potential for chronic and infected wounds. For instance, nanofiber–hydrogel composite dressings combining PLATMC nanofibers and GelMA hydrogels demonstrated antioxidant, antibacterial, and anti-inflammatory properties while promoting angiogenesis and collagen deposition in diabetic wound models [93]. Other systems, including WSCA-PNIPAM hydrogels and MXene-based hydrogel composites, have further improved hemostasis, antimicrobial activity, and tissue regeneration in preclinical studies [94,95,96].

#### 3.1.3. Enzyme-Responsive Hydrogels

Chronic wounds are frequently associated with elevated levels of proteolytic enzymes, including matrix metalloproteinases (MMPs), elastases, and collagenases, which contribute to excessive extracellular matrix degradation and delayed tissue repair [97,98]. Enzyme-responsive hydrogels are designed to exploit this biochemical imbalance by incorporating peptide sequences or chemical linkages that can be selectively cleaved by these enzymes [89]. When exposed to elevated enzymatic activity, the hydrogel network undergoes structural changes—such as crosslink cleavage, altered porosity, or accelerated degradation—resulting in the controlled release of encapsulated therapeutic agents [97,99,100].

This mechanism enables localized and self-regulated drug delivery, where release rates increase in response to disease severity and gradually decrease as the wound environment returns to physiological conditions [101]. In addition, the gradual degradation of the hydrogel matrix can facilitate cellular infiltration, extracellular matrix deposition, and vascularization, thereby supporting tissue regeneration [102].

Several enzyme-responsive hydrogel systems have demonstrated promising therapeutic potential in preclinical wound models. For example, hydrogels incorporating copper-based bioactive components or curcumin-loaded nanoparticles have exhibited strong antibacterial activity, reduced inflammation, and enhanced angiogenesis in infected wounds [103,104]. Other polyphenol-derived hydrogels have shown multifunctional properties—including adhesion, hemostasis, and photothermal antibacterial activity—resulting in accelerated healing in infected wound models [105].

#### 3.1.4. Light/Photocrosslinkable Hydrogels

Light-responsive hydrogels constitute an additional category of intelligent materials that can be externally activated by visible or ultraviolet light to initiate crosslinking, structural modifications, or the release of active agents [106]. These systems typically incorporate photoreactive compounds, such as light inducers or photo-cleavable crosslinkers, which undergo chemical transformations upon exposure to specific wavelengths [107]. This provides a superior degree of spatial and temporal precision, enabling clinicians to activate hydrogel solidification accurately at the incision site, even within geometrically intricate regions [108]. Photocurable hydrogels are especially advantageous for internal lesions, surgical incisions, and trauma events requiring prompt sealing and hemostasis [109]. By applying or distributing the hydrogel precursor across the lesion and subsequently administering localized light exposure, a stable and conformal gel barrier can be established within seconds [110]. This removes the necessity for sutures or mechanical fixation while establishing an occlusive environment that safeguards against bacterial infiltration [111]. Furthermore, due to the ability to spatially confine the crosslinking process, light-responsive hydrogels are compatible with advanced manufacturing techniques such as microfabrication and 3D printing, facilitating the creation of personalized wound dressings with adjustable architecture [112]. Their ability to undergo controllable transitions further enhances their potential as platforms for the precise release of drugs or bioactive molecules, where external light exposure can trigger pulsatile or on-demand therapeutic delivery [113].

Several photocrosslinkable hydrogel systems have demonstrated promising applications in wound repair. For example, GelMA hydrogels incorporating stem-cell-derived exosomes promoted endothelial cell proliferation, angiogenesis, and macrophage M2 polarization in full-thickness wound models [114]. Hybrid photocrosslinked SilMA–GelMA hydrogels have also shown improved mechanical strength, biodegradability, and enhanced collagen deposition in vivo [115]. Additional multifunctional photocurable systems incorporating antioxidant or photothermal components have further improved tissue regeneration and inflammation control in preclinical studies [116,117].

#### 3.1.5. Electrically Responsive Hydrogels

Electrical stimulation has been demonstrated to expedite wound healing by facilitating cellular migration, augmenting fibroblast as well as keratinocyte proliferation, inducing angiogenesis, and exerting direct antimicrobial actions [118]. Electrically responsive hydrogels integrate conductive materials, including graphene, polyaniline, polypyrrole, and carbon nanotubes, into their structures, enabling them to detect endogenous electrical activity or serve as conductive scaffolds that deliver therapeutic electrical signals to the wound site [89]. These hydrogels are capable of modifying their structural properties in response to an applied electric field, as electrostatic interactions within the matrix affect polymer conformation, swelling, and fluid transport [119]. The capacity to regulate swelling can be utilized for electrically controlled drug release, whereby ionic drugs or charged therapeutic molecules are driven from the hydrogel in response to precise electrical stimulation [87]. Electrically conductive hydrogels also establish dynamic microenvironments that facilitate intercellular communication and promote tissue regeneration, thereby serving as valuable components in innovative bioelectronic wound dressings [120]. Their incorporation into adaptable peripheral devices further facilitates real-time assessment of wound status and personalized therapeutic interventions. The versatile properties of electrically responsive hydrogels establish them as one of the most sophisticated platforms for next-generation wound management systems [121].

Recent studies have reported several electrically responsive hydrogel platforms capable of accelerating wound healing through electrostimulation and controlled drug delivery. For instance, triboelectroresponsive DNA hydrogels and piezoelectric nanocomposite hydrogels have demonstrated antibacterial activity, angiogenesis promotion, and enhanced tissue regeneration in infected or diabetic wound models [122]. Other conductive hydrogel systems, including electroresponsive e-Patch platforms and PEDOT-based hydrogels, have further improved fibroblast migration, electrical responsiveness, and wound closure in preclinical studies [123,124,125].

### 3.2. Injectable and Self-Healing Hydrogels

Injectable and self-healing hydrogels constitute significant advancements in wound management, as they enable minimally invasive administration and preserve structural integrity under mechanical stress resulting from joint movement or patient activity [126]. Injectable hydrogels are engineered to undergo shear thinning during administration and subsequently transform into a solid or semi-solid gel in situ, thereby obviating the necessity for surgical implantation. This in situ gelling behavior may be initiated by variations in temperature, pH, ion concentration, light exposure, or inherent shear-thinning properties [127]. When administered into deep, irregular, or difficult-to-reach wound cavities, injectable hydrogels precisely adapt to the surrounding tissue, establishing close contact that facilitates the healing process [128]. Their capacity to encapsulate cells, nanoparticles, growth factors, and medications ensures that therapeutic agents remain intact during administration and are effectively concentrated within the wound environment. Consequently, injectable hydrogels have emerged as valuable platforms for managing tunneling wounds, burns, pressure ulcers, and surgical cavities that require localized, long-term therapeutic intervention [129].

Self-healing hydrogels further enhance these benefits by integrating dynamic chemical interactions that restore structural integrity following mechanical damage. These interactions typically include reversible covalent bonds, metal–ligand coordination, hydrogen bonding, or host–guest complexation, enabling the polymer network to rapidly regenerate after disruption [130]. This self-repair capability is particularly important for wounds located on joints or other highly mobile body regions, where repeated stretching, compression, and shear forces may compromise conventional hydrogels [131]. Consequently, even when the dressing is punctured or mechanically disturbed, it can autonomously reseal without losing its protective function. In some systems, self-healing is combined with electrical conductivity, allowing simultaneous structural recovery and electrostimulation to promote tissue regeneration [106]. Collectively, these features make injectable and self-healing hydrogels promising platforms for clinically complex wounds where mechanical disturbance and patient mobility are significant considerations [132].

Recent preclinical studies have further demonstrated the therapeutic versatility of these systems. For example, Schiff-base-crosslinked and oxidized polysaccharide-based injectable hydrogels have shown strong adhesion, antioxidant, antibacterial, and immunomodulatory effects, leading to improved healing in burn and diabetic wound models [133,134,135]. Other multifunctional platforms, including silver nanoparticle-, Laponite-, and chitosan-based hydrogels, have also demonstrated enhanced hemostasis, infection control, angiogenesis, collagen deposition, and epithelial regeneration in irregular or infected wound models [136,137,138,139].

### 3.3. 3D-Printable and Shape-Adaptive Hydrogels

Advances in 3D printing and digital fabrication have significantly transformed biomaterial engineering, enabling personalized wound management through hydrogels fabricated to match the complex geometry of individual wounds [67]. 3D-printable hydrogels, commonly referred to as bioinks, are designed with rheological properties that allow smooth extrusion through printing nozzles while maintaining structural fidelity after deposition. These materials typically exhibit shear-thinning behavior, rapid structural recovery, and crosslinking mechanisms compatible with printing processes and biological components [140]. Many bioinks are derived from modified natural polymers such as gelatin methacryloyl, alginate composites, hyaluronic acid derivatives, and polyethylene glycol-based systems, which can be stabilized through ionic, thermal, or photo-induced crosslinking [141]. Their ability to incorporate living cells, biomolecules, and therapeutic agents enables the fabrication of multifunctional wound dressings capable of promoting tissue regeneration and localized drug delivery [142,143,144,145].

Shape-adaptive hydrogels represent another class of smart materials capable of dynamically conforming to wound geometry or altering their configuration in response to external stimuli [146]. These materials may exhibit shape memory, elasticity, or responsive swelling behavior that allows them to fill irregular wound cavities and maintain close contact with surrounding tissues [147]. Some systems are engineered to maintain a temporary configuration at low temperatures and transform into a stable structure at physiological conditions, facilitating convenient application and improved wound coverage [148]. Such adaptive behavior is particularly beneficial for wounds located near joints or other mobile anatomical regions, where conventional dressings may become displaced [120]. In addition to improving mechanical protection, shape-adaptive hydrogels can enhance patient comfort and reduce the frequency of dressing changes in chronic wound care [149].

The design of hydrogel bioinks requires a careful balance between printability and biological compatibility. High viscosity is necessary to maintain filament stability during extrusion-based printing; however, excessive viscosity may generate shear stress that compromises cell viability [150,151]. To address this challenge, most bioinks are engineered to exhibit shear-thinning behavior, allowing viscosity to decrease during extrusion and rapidly recover after deposition. Rheological modifiers such as nanoclay, cellulose nanofibers, and gelatin derivatives are often incorporated to improve printability and structural stability. Rapid and controlled crosslinking mechanisms—including ionic, photo-induced, enzymatic, and thermal gelation—are commonly employed to stabilize printed constructs and maintain mechanical integrity [152,153,154,155,156,157].

Recent preclinical studies have demonstrated the potential of 3D-printed hydrogel systems in wound repair. For example, nanoclay-reinforced chitosan hydrogels containing halloysite nanotubes have shown improved mechanical strength and sustained antibacterial drug release, leading to accelerated healing in infected wound models [158]. Other printable hydrogel systems, including gelatin/κ-carrageenan dual-network hydrogels and photocurable gelatin-based formulations, have demonstrated enhanced structural stability, biocompatibility, antioxidant activity, and improved tissue regeneration in animal models [159,160].

## 4. Functionalized Hydrogels for Therapeutic Enhancement

To address the escalating prevalence of chronic wounds, functionalized hydrogels have emerged as versatile therapeutic platforms offering antibacterial activity, pro-angiogenic support, and enhanced tissue regeneration [161]. Recent literature continuously emphasizes the potential of these advanced dressings to overcome challenges associated with a diverse array of wound types, particularly diabetic ulcers [162]. By providing precise, stimuli-responsive drug delivery alongside anti-inflammatory and antimicrobial properties, functional hydrogels offer substantial therapeutic benefits, notably in the management of complex oral and maxillofacial wounds. These versatile systems actively facilitate all phases of tissue regeneration by serving as efficient carriers for a wide variety of therapeutic cargos [163]. Furthermore, naturally derived biopolymers—such as chitosan, alginate, and cellulose—are frequently utilized to fabricate biocompatible hydrogel dressings that promote optimal healing by maintaining a moist microenvironment and preventing infection. Modern fabrication techniques now enable the production of highly customized dressings, while sophisticated strategies, including targeted chemical modifications, bioactive agent integration, and stimuli-responsive engineering, continue to profoundly enhance their overall therapeutic efficacy [164].

Despite the substantial advancements achieved in laboratory settings, a critical evaluation of the current landscape reveals that the direct clinical application of functionalized hydrogels in human patients remains limited. The vast majority of existing literature is predicated on in vitro assessments, accompanied by relatively few in vivo animal models and a notable scarcity of rigorous clinical validation [165]. Consequently, the healing outcomes and therapeutic efficacies discussed in this context are predominantly derived from experimental and preclinical models rather than established clinical data. Acknowledging this significant translational gap, future research must prioritize comprehensive, carefully designed clinical trials to rigorously evaluate the long-term safety, clinical efficacy, and practical utility of these advanced hydrogel systems in real-world wound care settings [112].

### 4.1. Antimicrobial Hydrogels

Targeted drug delivery is gaining critical importance due to the escalating prevalence of antibiotic-resistant microorganisms [166]. The hydrophilic, three-dimensional networks of biodegradable hydrogels allow for the controlled, prolonged release of antimicrobial agents, making them highly suitable carriers [167]. Recent literature emphasizes various types of hydrogels, detailing their preparation techniques, mechanisms of action, and clinical applications, with a particular focus on metal nanoparticle-loaded hydrogels as a potent resistance-fighting strategy [168]. Furthermore, antimicrobial hydrogels are emerging as promising therapeutics for dental tissue infections owing to their excellent water retention, biocompatibility, and capacity to effectively deliver medications or nanoparticles. Despite existing challenges related to implantation, sophisticated 3D and 4D printing technologies offer future avenues for innovation in these smart, multifunctional biomaterials, aiming to simultaneously boost tissue regeneration and combat infection [169]. Additionally, antimicrobial peptide–hydrogel associations (PHAs) have been reported as a viable substitute for conventional antibiotics in the global fight against multidrug-resistant bacteria (MDRB). PHAs demonstrate robust efficacy against MDRB while offering promise as versatile, responsive antimicrobial systems by improving peptide stability, biological compatibility, and targeted transport [170].

**Figure 2:**
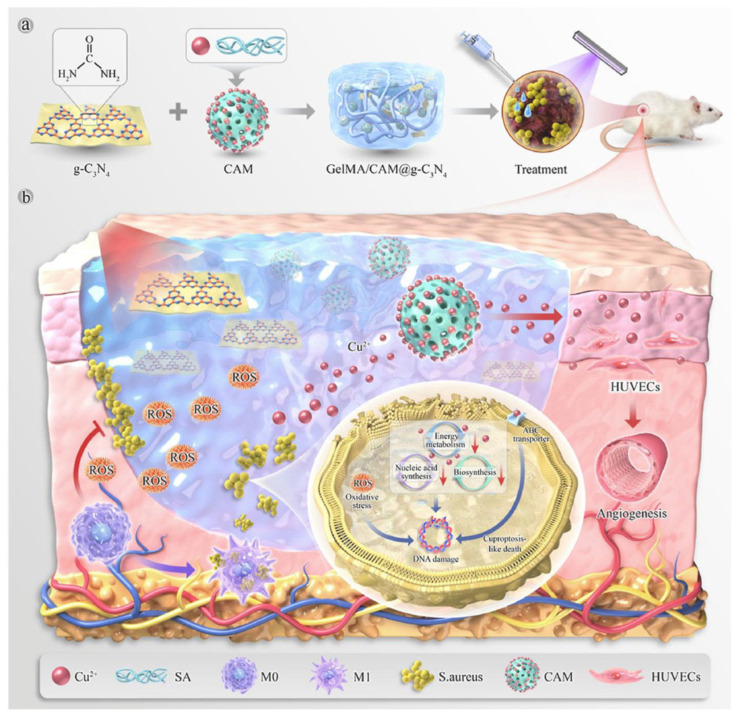
Depiction of (**a**) formulation of g-C_3_N_4_ based hydrogel microspheres; (**b**) macrophage M1 reactivation and sustained copper ion release provide continuous antibacterial action, promote angiogenesis, and induce copper-mediated bacterial death. Reproduced with permission under CC BY-NC-ND 4.0 license from ref. [171].

**Figure 3:**
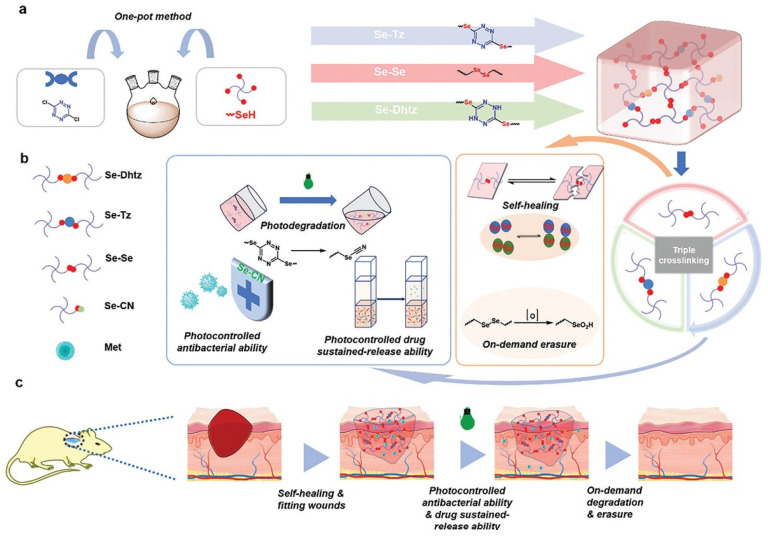
Synthesis, properties, and in vivo applications of the multiple-crosslinked Se-Tz hydrogel. (**a**) Schematic of the one-pot, environmentally friendly synthesis approach, yielding a hydrogel with three chemical cross-linking mechanisms (diselenide, aryl selenide, and dearomatized selenide bonds). (**b**) Illustration of the hydrogel’s unique property profile, demonstrating its capacity for photodegradation, photocontrolled drug sustained-release, photocontrolled antibacterial ability, self-healing, and on-demand erasure. (**c**) Animal studies demonstrating the hydrogel’s practical applications in wound care, including self-healing and wound fitting, light-triggered antibacterial and drug release activities, and on-demand degradation. Reproduced with permission under the CC BY 4.0 license from ref. [172].

As summarized in Table 2, smart hydrogel technologies present a wide array of functional strategies for wound management, encompassing various hydrogel matrices, functional components, mechanisms of action, and biological effects. In this context, Xiang et al. developed a straightforward method for fabricating multi-responsive, on-demand degradable selenol–tetrazine (Se–Tz) hydrogels utilizing mild reactions without the necessity for additives or complicated processing. In vivo evaluations confirmed improved healing in diabetic wounds, successfully demonstrating the hydrogels’ intrinsic self-healing capacity, regulated degradation, light-induced antibacterial activity, and efficient drug administration (Figure 2) [171]. Similarly, Wu et al. introduced an additive-free technique to create dynamic, rapidly degrading Se–Tz hydrogels featuring light-activated antibacterial and self-healing capabilities (Figure 3). The integration of metformin into this system resulted in highly effective diabetic wound healing by further increasing angiogenesis and collagen deposition. Owing to their robust mechanical properties, significant antibacterial activity, and controlled breakdown, these hydrogels represent a highly viable substrate for advanced wound dressings [172].

### 4.2. Drug- and Growth-Factor-Loaded Systems

The development of bioactive hydrogels (Bio-HyGs)—such as those formulated from gelatin methacryloyl (GM), polyethylene glycol (PEG), and polyvinyl alcohol (PVA)—has been widely reported for controlled therapeutic delivery and wound healing, particularly in the context of chronic wounds. These hydrogels are specifically engineered for the controlled and sustained release of bioactive molecules, including vascular endothelial growth factor (VEGF) and platelet-derived growth factor (PDGF), thereby facilitating tissue regeneration without the prerequisite of initial cell seeding. Furthermore, recent technological advancements, including 3D printing, self-assembling peptides, and thermoresponsive polymer networks, significantly enhance tissue biomimicry. This structural and functional mimicry of the native extracellular matrix facilitates more efficient healing and superior therapeutic outcomes [19].

**Figure 4:**
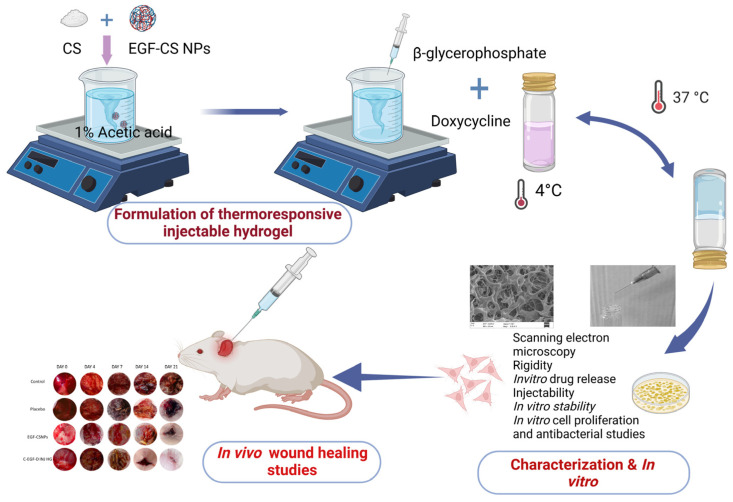
Schematic illustration of the design of a heat-sensitive hydrogel loaded with growth factors for improved diabetic wound healing. Reproduced with permission under the CC BY 4.0 license from ref. [173].

To specifically address the prolonged healing process of diabetic wounds, Tallapaneni et al. engineered an injectable, thermoresponsive hydrogel incorporating doxycycline alongside epidermal growth factor (EGF)-loaded chitosan nanoparticles (Figure 4). This advanced formulation demonstrated excellent biocompatibility, robust antibacterial activity, sustained drug release profiles, and highly favorable physicochemical characteristics. In vivo evaluations revealed that the hydrogel accelerated all phases of the wound healing cascade by significantly mitigating inflammation, promoting robust collagen synthesis, and driving rapid epithelial regeneration. Collectively, these findings underscore the substantial clinical potential of this dual-loaded system as a highly effective therapeutic intervention for diabetic wounds [173].

### 4.3. Cell-Encapsulated and Tissue-Engineering Platforms

Numerous cell-engineered technologies for tissue regeneration and wound healing have been developed, utilizing various cell types that can be further genetically modified to enhance their therapeutic efficacy [174]. Additionally, cutting-edge cell delivery strategies, including 3D bioprinting and sophisticated hydrogel networks, have been extensively investigated to improve their clinical applicability [175,176]. Advanced cell encapsulation strategies have been strategically employed to promote tissue regeneration by leveraging dynamic immunological niche interactions. To optimize these microenvironments, innovative techniques are continuously being integrated into the design of cell encapsulation formulations, encompassing layer-by-layer assembly, microfluidics, superhydrophobic coatings, bioprinting, and modular hydrogel-based systems [177].

**Figure 5:**
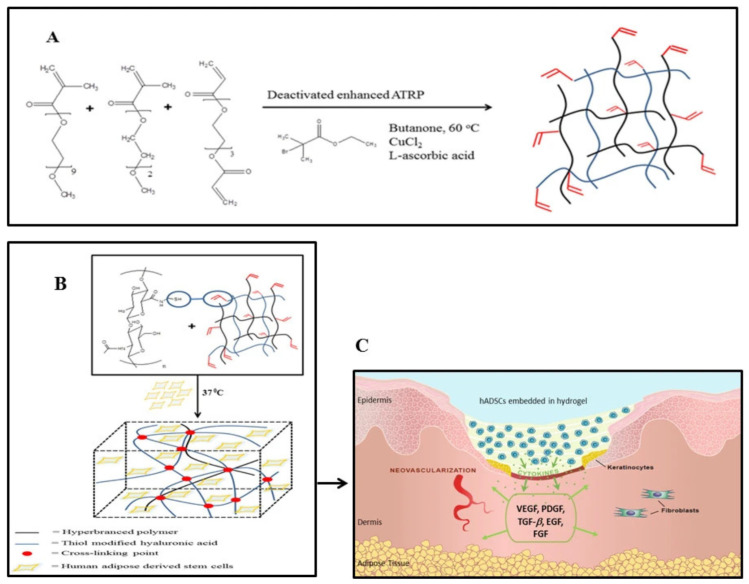
Schematic illustration of (**A**) the structural scheme of copolymer synthesis via ATRP, (**B**) the Michael-type crosslinking using thiolated hyaluronic acid for encapsulating adipose-derived stem cells, and (**C**) the subsequent hydrogel applied to wounds to deliver regenerative growth factors generated by stem cells. Reproduced with permission under the CC BY 2.0 license from ref. [178].

In a notable application of this approach, Wang et al. evaluated adipose-derived stem cells as a potential living wound dressing by encapsulating them within a thermoresponsive polyethylene glycol (PEG)–hyaluronic acid-based hydrogel (Figure 5). Over a seven-day observation period, this system exhibited reduced cellular proliferation but significantly increased the localized release of essential pro-angiogenic factors, such as VEGF and PlGF; concurrently, the secretion profiles of inflammatory cytokines remained unaltered. The platform consistently maintained stable cellular functionality and demonstrated minimal cytotoxicity. Consequently, this thermoresponsive hydrogel exhibits substantial promise as a versatile, highly effective living substrate for advanced regenerative wound healing applications [178].

### 4.4. Nanocomposite and Multifunctional Hydrogels

To address the urgent need for effective clinical interventions for skin injuries caused by trauma, infection, or surgery, nanocomposite biopolymer hydrogels have emerged as versatile and efficient wound dressings. By incorporating nanoparticles within polymer matrices, these systems exhibit enhanced antimicrobial, antioxidant, anti-inflammatory, conductive, and mechanical properties, which collectively contribute to accelerated wound healing [179]. In addition, the integration of stimuli-responsive nanomaterials enables these hydrogels to respond to external physical fields, allowing on-demand therapeutic interventions and improved adaptability to dynamic wound environments [107].

Recent studies have demonstrated the significant therapeutic potential of nanocomposite hydrogels in wound repair. For instance, nanocomposite systems incorporating insulin-loaded chitosan nanoparticles and ciprofloxacin within alginate matrices have shown improved mechanical stability, high cytocompatibility, and controlled therapeutic release profiles that support multiple stages of the healing process [180]. Similarly, hydrogels containing hydrogen-capped Au–Pd nanoparticles with antisense oligonucleotides have exhibited strong antibacterial activity and accelerated wound healing in infected models, achieving up to 99% wound closure under near-infrared stimulation [181]. Additional multifunctional nanocomposite platforms have also been reported to enhance antibacterial performance and tissue regeneration in various wound healing models [182].

**Figure 6:**
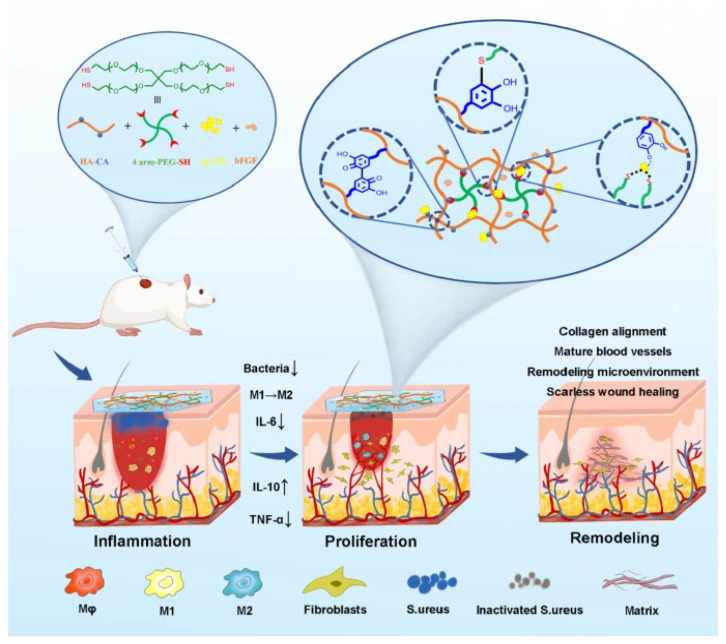
Schematic illustration of silver nanocomposite hydrogels designed to regulate the immune and regenerative microenvironment in infected wounds, enabling scarless wound healing. Reproduced with permission under the CC BY 4.0 license from ref. [184].

Further advancements include the development of nanocomposite hydrogels integrating tannin/siRNA nanogels with pH indicators, enabling simultaneous therapeutic intervention and real-time monitoring of the wound microenvironment. Such systems have demonstrated immunomodulatory effects, enhanced vascular regeneration, and improved antibacterial and antioxidant activities in infected or diabetic wound models [183]. Moreover, silver nanoparticle-based hydrogels have been engineered to achieve sustained release of growth factors such as basic fibroblast growth factor (bFGF), promoting macrophage polarization toward a pro-healing phenotype while enhancing collagen organization and vascularization (Figure 6) [184].

While metal-based nanocomposite hydrogels—particularly those containing copper, silver, and gold–palladium nanoparticles—exhibit remarkable antibacterial and pro-angiogenic properties, their therapeutic utility is often limited by dose-dependent cytotoxicity. Excessive local concentrations of heavy metals can impair tissue regeneration, diminish cell viability, and inhibit fibroblast proliferation. Therefore, rigorous toxicity evaluations must be established to guarantee safe clinical application [185]. A delicate equilibrium must be achieved between attaining potent antimicrobial efficacy—which frequently requires an initial burst release of metal ions—and maintaining long-term cytocompatibility to support tissue repair. This balance can be optimized by precisely calibrating metal concentrations, ensuring uniform nanoparticle dispersion within the hydrogel matrix, and strictly controlling release kinetics to prevent excessive localized accumulation [186]. By methodically addressing these parameters, researchers can engineer nanocomposite hydrogels that maximize infection control while minimizing cellular toxicity, ensuring both safety and therapeutic efficacy in preclinical models and future clinical translations [187].

### 4.5. Conductive Hydrogels for Accelerated Healing

Conductive hydrogels offer significant advantages for chronic wound healing by promoting cell migration, mitigating inflammation and infection, enhancing collagen synthesis and angiogenesis, and facilitating real-time monitoring [188]. These platforms provide sophisticated functionalities, including integrated diagnostics, electrically triggered drug delivery, and self-powered operation. With the future integration of artificial intelligence (AI), these systems possess the potential to revolutionize intelligent, personalized wound care [189]. In a notable advancement, Wang et al. developed an adjustable electronic patch (ePatch) that delivers efficient and adaptable electrical stimulation for wound healing, utilizing a conductive hydrogel composed of methacrylated alginate and silver nanowires. This formulation exhibits high clinical compatibility and potent antibacterial activity, enabling bespoke bioprinting onto medical-grade substrates. In vivo evaluations demonstrated that the ePatch significantly expedited healing in a rat model, achieving complete wound closure in 7 days compared to the typical 20 days. Furthermore, in vitro studies confirmed enhanced growth factor release, cell proliferation, and migration, collectively highlighting its tremendous promise for personalized wound management [190].

Leveraging dynamic noncovalent interactions, Ren et al. engineered a multifaceted ionic conducting hydrogel (HPMC/AA/AM/TP/Al^3+^) exhibiting skin-mimetic characteristics, including exceptional softness, stretchability, and robust tissue adhesion. The hydrogel demonstrated high conductivity, rapid electrical self-healing, and outstanding mechanical properties. When utilized as an epidermal sensor, it exhibited excellent sensitivity and rapid response times, successfully detecting subtle speech vibrations and gross joint movements. Consequently, this “all-in-one” hydrogel holds significant promise for future flexible, wearable sensing systems [191]. Similarly, Zhu and colleagues investigated a multipurpose conductive hydrogel dressing (GelMA@Ti_3_C_2_/V-Os) by integrating GelMA, conductive Ti_3_C_2_, and collagen-binding antimicrobial peptides to optimize electrical stimulation-based wound therapy. The resulting dressing demonstrated high electrical conductivity, excellent biocompatibility, and persistent antibacterial action. Under electrical stimulation, it significantly promoted angiogenesis, re-epithelialization, immunomodulation, and infection control, while simultaneously enhancing fibroblast migration, proliferation, and the expression of repair-related genes. This study presents a highly viable strategy for accelerating the healing of infected wounds [192].

Recent reports indicate that conductive hydrogels can synergize advanced material selection, sensor technology, and AI algorithms to predict healing trajectories using real-time, sensor-derived data. Their dual pharmacological and diagnostic utility has been successfully demonstrated in the advanced management of pressure ulcers, diabetic foot ulcers, and highly mobile joint wounds [193]. Expanding upon the development of functional hydrogels—encompassing conductive, hemostatic, antimicrobial, anti-inflammatory, and antioxidant variants—researchers have also reported the integration of conducting hydrogels with flexible solar cells to engineer carbon-neutral, self-powered wound dressings. These autonomous devices deliver continuous electrical stimulation to promote angiogenesis, cellular migration, and accelerated healing, while simultaneously minimizing maintenance and external energy requirements [194].

While functionalized hydrogels exhibit immense potential for wound management, it is crucial to recognize that the complex biochemical microenvironments of acute and chronic wounds remain insufficiently understood to enable the development of flawlessly accurate, individually “programmed” hydrogel systems [195]. Wound microenvironments are inherently dynamic and highly heterogeneous, fluctuating continuously based on the patient’s underlying clinical pathology, the severity and phase of infection, and the specific stage of healing [196]. Localized concentrations of proteolytic enzymes, inflammatory mediators, microbial populations, blood plasma components, and products of extracellular matrix degradation can vary significantly among different wounds [197]. This biological intricacy is further exacerbated in chronic wounds—such as those associated with vascular insufficiency, diabetes, or immunological dysfunction—due to the presence of recalcitrant microbial biofilms, delayed tissue regeneration, and sustained chronic inflammation [198]. Consequently, the precise biochemical composition and spatiotemporal evolution of the wound environment remain only partially elucidated. This profound heterogeneity severely hampers the rational design of functionalized hydrogels intended to dynamically react to, or precisely modulate, specific localized wound conditions. Therefore, acquiring a more comprehensive understanding of wound biochemistry and its patient-specific variability is imperative to ensure the reliable clinical translation of fully programmable and personalized hydrogel platforms [199,200].

Furthermore, despite the substantial advancements in hydrogel-based wound dressings, it is vital to acknowledge specific limitations when evaluating current preclinical findings. A significant portion of the mechanistic insights regarding hydrogel-mediated wound healing is derived from oversimplified experimental models, particularly 2D cell culture systems, which inherently fail to replicate the intricate biochemical signaling and complex 3D architecture of native human skin [201]. While animal models provide crucial preliminary data regarding biological responses and material biocompatibility, the human wound microenvironment remains notoriously difficult to faithfully replicate in vivo, particularly in the context of chronic pathologies such as diabetic ulcers, pressure ulcers, and ischemic wounds. Fundamental interspecies differences in immune responses, anatomical skin structure, microbial colonization patterns, and healing kinetics frequently lead to significant disparities between preclinical outcomes and actual clinical performance [202]. Consequently, the clinical translation of numerous promising hydrogel systems that exhibit exceptional therapeutic efficacy in vitro or in animal studies often encounters substantial translational roadblocks. To accurately evaluate the efficacy, practical feasibility, and long-term safety of next-generation hydrogel dressings, future research must pivot toward the development of highly sophisticated experimental platforms, including 3D human skin equivalents, organ-on-a-chip wound models, and rigorously designed human clinical trials [203].

In conclusion, the diverse functionalized hydrogel platforms discussed throughout this section demonstrate immense potential in addressing the multifaceted challenges of wound healing. To concisely summarize these advancements, Table 2 highlights selected representative examples of key smart hydrogel technologies, detailing their primary functional components, underlying mechanisms of action, and specific biological effects across various applications.

**Table 2:** Selected representative examples of smart hydrogel technologies and their biological functions in wound healing.

Hydrogel Type	Functional Component	Key Mechanism	Biological Effect	Ref.
Antimicrobial hydrogel	Se–Tz network	Light-triggered antibacterial activity	Diabetic wound healing	[171]
Growth factor hydrogel	EGF-loaded thermoresponsive hydrogel	Sustained growth factor release	Accelerated diabetic wound repair	[173]
Cell-encapsulation hydrogel	PEG-HA hydrogel + adipose-derived stem cells	Growth factor secretion	Enhanced angiogenesis and tissue regeneration	[178]
Nanocomposite hydrogel	Hydrogen-capped Au–Pd nanoparticles	Photothermal antibacterial action	Rapid infected wound healing	[181]
Conductive hydrogel	Alginate + silver nanowires	Electrical stimulation	Accelerated wound closure	[190]

## 5. Characterization and Evaluation of Smart and Functionalized Hydrogels

The functionalization of hydrogels—achieved through the integration of antimicrobial agents, growth factors, nanoparticles, natural extracts, peptides, or bioactive polymers—significantly augments their therapeutic efficacy. These advanced platforms actively enhance fibroblast proliferation, collagen deposition, angiogenesis, and rapid re-epithelialization. Furthermore, the incorporation of antimicrobial and antibiofilm agents is crucial for preventing infection, which remains a significant impediment to successful wound healing.

The escalating clinical interest in bioengineered wound dressings necessitates the rigorous characterization and evaluation of these smart, functionalized hydrogels. Physicochemical investigations are essential to elucidate swelling behavior, network architecture, degradation kinetics, and mechanical integrity. Concurrently, comprehensive biocompatibility and antimicrobial evaluations ascertain both safety and therapeutic effectiveness, while in vivo wound-healing models provide definitive evidence of their regenerative capacity [204,205]. Collectively, these synergistic methodologies facilitate the systematic design of advanced hydrogel systems capable of expediting tissue repair, mitigating clinical complications, and ultimately enhancing patient outcomes.

### 5.1. Physicochemical and Mechanical Testing

Comprehensive characterization of smart hydrogels involves a multi-modal approach to evaluate their structural, morphological, rheological, and mechanical properties.

#### 5.1.1. Structural and Compositional Analysis

- X-Ray Diffraction (XRD), Thermal, and FTIR Analysis; XRD is utilized to confirm the crystallinity or amorphous nature of the hydrogel components. Concurrently, Differential Scanning Calorimetry (DSC) and Thermogravimetric Analysis (TGA) assess thermal stability and phase transitions. Fourier Transform Infrared (FTIR) spectroscopy is subsequently employed to confirm polymer–drug interactions and validate the successful functionalization of the polymeric network [206].
- Nuclear Magnetic Resonance (NMR) Spectroscopy: Solid-state NMR, particularly magic-angle spinning (MAS) techniques like ^1^H–^13^C CP-MAS, elucidates the molecular structure in the solid-like gel phase. These studies reveal network rigidity (e.g., phenylalanine and tyrosine forming rigid networks, while leucine and serine remain mobile). Solution-state, variable-temperature, and saturation transfer difference (STD) NMR provide critical insights into gelation dynamics, molecular uptake, and ligand binding processes [207].
- Circular Dichroism (CD): CD spectroscopy is used to analyze conformational changes and secondary structures in hydrogel networks. For example, studies on Fmoc-protected amino acid hydrogels have confirmed the presence of π–π interactions and helical structures, where peak shifts correlate with variations in ionic strength and network rigidity [208].
- X-Ray Scattering (WAXD/SAXS): Wide-Angle X-ray Diffraction (WAXD) and Small-Angle X-ray Scattering (SAXS) are utilized to precisely visualize macromolecular sizes, shapes, and structural organization within supramolecular hydrogels [209,210].

#### 5.1.2. Morphological Characterization

- Electron Microscopy (SEM, TEM, and Cryo-EM): Scanning Electron Microscopy (SEM) evaluates physical characteristics such as pore dimensions, gel porosity, and polymeric strand orientation following cryogenic preservation. Transmission Electron Microscopy (TEM) visualizes nanoscale features like fibrils and micelles. Cryo-EM maintains the native hydrated state of the sample, significantly reducing drying artifacts. Together, these techniques offer profound insights into the micro- and nanostructural arrays of the matrices [211].

#### 5.1.3. Rheological Properties

- Shear-Thinning and Self-Healing: Crucial for 3D bioprinting and injectable drug delivery, shear-thinning behavior occurs when applied shear stress disrupts the supramolecular network, leading to a reversible reduction in viscosity [212].
- Strain Sweep Testing: This amplitude sweep test evaluates viscoelasticity by applying an increasing oscillatory strain at a constant frequency. It measures the storage modulus (G′) and loss modulus (G″). A constant modulus defines the linear viscoelastic region (LVR), while a G′–G″ crossover indicates the gel–sol transition, marking the point where the material begins to exhibit fluid-like behavior [213,214].

#### 5.1.4. Physical and Mechanical Evaluations

- Swelling Index: This critical parameter quantifies the hydrogel’s capacity to absorb wound exudate prior to matrix degradation [215].
- Gel Fraction and Crosslinking Effects: Three-dimensional networks are established via chemical crosslinking, physical linkages, ionic interactions, or hydrogen bonding. While synthetic chemical crosslinkers (e.g., epichlorohydrin, aldehydes, urea derivatives) efficiently interconnect polymer chains, unreacted reagents can be hazardous. Therefore, meticulous post-crosslinking purification or the adoption of green crosslinking strategies is imperative to ensure biocompatibility and environmental sustainability [216].
- Degradation and Erosion Studies: Custom-fabricated cast acrylic molds are frequently employed to standardize hydrogel geometry for erosion studies. Hydrogels—often fluorescently labeled or loaded with a model biomolecule like FITC–BSA (0.1 wt%)—are exposed to a buffer reservoir (e.g., PBS) under static conditions for extended periods (up to 60 days). Regular colorimetric quantification yields cumulative erosion and release profiles, providing vital insights into composition-dependent degradation kinetics [217].
- Mechanical Testing: Typically assessed using a universal testing machine in accordance with ASTM D638 standards, mechanical qualities such as tensile strength, fracture energy, and elastic modulus are evaluated. A hydrogel’s mechanical response to applied stress is a highly reliable predictor of its in vivo durability and tissue regeneration efficacy [218].

### 5.2. Antimicrobial and Biocompatibility Studies

To ensure reproducibility during characterization, hydrogels are typically fabricated using standardized molds, guaranteeing uniform geometry and volume across all samples. Following preparation, the constructs are incubated in controlled physiological buffer systems to rigorously monitor their swelling kinetics, structural degradation, and the release profiles of any embedded therapeutic agents over time. To facilitate precise quantification of these dynamic processes, fluorescent dyes or model biomolecules are frequently utilized; aliquots of the release medium are sequentially sampled at predetermined intervals and analyzed using standard laboratory techniques.

Parallel to these physicochemical assessments, the biological safety of the hydrogel platforms must be meticulously validated. In vitro cytocompatibility is conventionally evaluated by exposing relevant skin-associated cell lines, such as primary fibroblasts or keratinocytes, to hydrogel extracts, followed by quantitative assessments of cellular viability and proliferation. Furthermore, hemocompatibility is strictly evaluated by incubating the extracts with red blood cells to verify that the material does not induce hemolysis. When necessary, this is supplemented by standard coagulation cascades or platelet interaction assays to ensure complete blood compatibility.

Given the heightened susceptibility of open wound microenvironments to bacterial colonization, evaluating antimicrobial efficacy is an equally critical requirement. Hydrogel samples are subjected to established microbiological assays—such as being positioned in direct proximity to prevalent wound-associated pathogens—where the degree of microbial suppression is systematically documented on agar plates or within liquid broth cultures. Collectively, these indispensable evaluations substantiate the physical stability of the hydrogel, its excellent biocompatibility with living tissues, and its robust capacity to maintain a sterile, infection-free environment conducive to optimal wound healing [219,220].

### 5.3. In Vivo Studies

In vivo wound-healing evaluations predominantly utilize healthy laboratory rodents, such as rats, maintained under standardized housing conditions and strict ethical oversight. Following the administration of appropriate anesthesia, the dorsal region is shaved, sterilely prepped, and precise surgical incisions are made to create subcutaneous pockets. The test and control hydrogel formulations are subsequently implanted into these sites. For investigations requiring an infection model, a precisely quantified volume of bacterial suspension (or saline for controls) is inoculated into the pocket prior to wound closure via suturing. Postoperatively, the animals receive appropriate analgesia and are meticulously monitored throughout the predefined study duration. At specified time points, the subjects are humanely euthanized; the sutures are removed, and the implanted hydrogels, alongside the surrounding tissues and wound exudates, are carefully explanted. These retrieved samples undergo rigorous histological and biochemical evaluations to comprehensively assess infection control, host tissue response, and overall wound healing efficacy [220].

Furthermore, in vivo fluorescence imaging serves as a highly sensitive, non-invasive modality for visualizing dynamic biological processes within living organisms using specialized fluorescent probes. This technique relies on the excitation of fluorophores via an external light source, followed by the capture of the emitted photons using advanced optical imaging systems. This sophisticated approach enables the real-time spatiotemporal tracking of molecular events, disease progression, and localized drug delivery within living tissues. While extensively applied in preclinical animal research—particularly for oncological imaging and therapeutic efficacy assessments—its clinical utility is most prominently demonstrated in image-guided surgery and angiography utilizing approved fluorescent dyes, such as indocyanine green (ICG) [221].

## 6. Clinical Applications and Commercial Outlook

Functionalized hydrogels have emerged as a revolutionary class of biomaterials for wound care, owing to their excellent biocompatibility, customizable physicochemical properties, and capacity to integrate diverse therapeutic agents, including growth factors, antimicrobials, nanoparticles, and living cells [222]. The majority of currently available clinical products are hydrogel-based dressings approved for the management of both acute and chronic wounds. These primarily include hydrogel-impregnated gauzes, sheet hydrogels, and amorphous hydrogels [223]. Formulated from biomaterials such as collagen, hyaluronic acid, polyethylene glycol (PEG), polyvinyl alcohol (PVA), and alginate, these products are frequently utilized to promote autolytic debridement, maintain a moist wound environment, and alleviate pain [224]. While many commercially available hydrogels function merely as passive dressings, more recent and sophisticated functionalized platforms—incorporating silver ions, antimicrobial peptides, bioadhesive chemistries, and controlled-release systems—have recently entered the initial phases of clinical testing [225]. However, the vast majority of multifunctional and intelligent hydrogels—such as those incorporating biosensors, gene carriers, or cell-laden matrices—remain in the preclinical or early translational stages [226].

State-of-the-art passive commercial hydrogel dressings serve as essential clinical benchmarks due to their established efficacy in wound care. These hydrogels, particularly those based on carboxymethylcellulose or crosslinked PVA, facilitate autolytic debridement, maintain optimal wound moisture, and provide both pain relief and thermal insulation [227]. To further optimize these dressings, researchers frequently incorporate highly absorbent layers or antimicrobial treatments to manage excess exudate and mitigate the risk of infection [228]. Possessing well-defined safety profiles and established regulatory approval, these commercial products act as ideal control standards when assessing novel hydrogel technologies. This allows researchers to benchmark advancements in healing kinetics, biocompatibility, and bioactive delivery efficacy against proven clinical standards without the strict necessity for parallel active-trial data [229].

Unfortunately, stringent regulatory considerations have significantly limited the market penetration of advanced functionalized hydrogels. Conventional passive hydrogels typically meet the standard regulatory requirements for medical devices, which primarily focus on basic performance, safety, and biocompatibility [230]. Conversely, multifunctional hydrogels incorporating active pharmaceutical ingredients, nanoparticles, or biologics are often classified as combination products. This classification necessitates far more rigorous regulatory scrutiny, demanding extensive data on pharmacokinetics, long-term toxicity, and precise mechanisms of action [231]. Furthermore, cell-laden or gene-modulating hydrogels face even steeper regulatory hurdles due to profound concerns regarding immunogenicity, genetic stability, and unpredictable in vivo behavior [199]. The path to regulatory approval is further complicated by the stringent requirements to ensure batch-to-batch consistency, validate sterilization protocols without degrading bioactivity, and demonstrate scalable manufacturing capabilities [232].

Ultimately, market adoption is heavily influenced by the hydrogel’s ease of use in clinical settings, its overall production costs, and its ability to demonstrate clear, distinct therapeutic advantages over conventional dressings [233,234]. Although the global wound care market is rapidly expanding—driven by aging populations and an increasing prevalence of diabetes and chronic wounds—the commercialization of advanced functionalized hydrogels remains hampered by exorbitant development costs, restrictive reimbursement frameworks, and the necessity for robust, large-scale clinical evidence [235]. Consequently, the future commercial success of next-generation functionalized hydrogels will depend critically on continuous advancements in scalable manufacturing technologies, proactive alignment with regulatory pathways, and rigorous practical validation in clinical settings [236].

## 7. Challenges and Future Perspectives

### 7.1. Manufacturing and Scalability Bottlenecks

Despite remarkable advances in their design and therapeutic efficacy, large-scale production and scalability remain the primary bottlenecks hindering the clinical translation of smart hydrogels. Within academic settings, hydrogel synthesis typically relies on highly controlled laboratory conditions, analytical-grade reagents, and customized crosslinking protocols that are not readily transferable to industrial manufacturing environments. Consequently, batch-to-batch variability, polymer heterogeneity, insufficient standardization of rheological properties, and fluctuating degradation profiles severely undermine reproducibility during scale-up. Furthermore, the incorporation of sensitive biological agents—such as growth factors, peptides, nanoparticles, and living cells—introduces substantial manufacturing complexity. This necessitates stringent aseptic processing, cold-chain logistics, and rigorous adherence to complex regulatory frameworks. The critical need for terminal sterilization techniques that preserve both the mechanical integrity and bioactivity of the hydrogel further complicates industrial feasibility, as conventional treatments like autoclaving or gamma irradiation frequently denature these sensitive bioactive components. Therefore, establishing cost-effective production pipelines, continuous synthesis methodologies, and scalable 3D bioprinting or extrusion-based manufacturing processes remains a formidable challenge.

### 7.2. Technological Hurdles in Sensor Hybridization

Another significant barrier lies in the seamless integration of hydrogel matrices with biosensors, flexible electronics, and smart bandage platforms. Although hydrogels offer superior moisture retention, breathability, and tunable drug-loading capacities, embedding microelectronics or biochemical sensing modules without compromising the material’s inherent flexibility or swelling kinetics presents profound technological hurdles. Complex challenges emerge regarding sustained power supply, mechanical strain mismatch at the hydrogel–sensor interface, signal degradation secondary to fluid penetration, and rapid sensor biofouling upon prolonged exposure to protein-rich wound exudates. Furthermore, the very nature of biodegradable hydrogels limits sensor longevity, as continuous dissolution or enzymatic degradation risks exposing delicate microcircuits directly to the corrosive wound microenvironment. To maintain absolute biocompatibility and prevent the release of harmful leachables, advanced interfacial engineering, the strategic integration of conductive polymers, and fail-safe encapsulation techniques are imperative to ensure the synchronous functioning of tissue-interfacing hydrogels and their embedded electronic modules. Additionally, while continuous real-time monitoring generates substantial physiological data streams, the infrastructure for effective data management, secure transmission, and actionable clinical interpretation remains vastly underdeveloped.

### 7.3. AI Integration and Next-Generation Autonomous Platforms

The prospective integration of artificial intelligence (AI)-driven wound monitoring introduces further complexities. Training robust machine learning algorithms requires massive, highly annotated clinical datasets, the acquisition of which is heavily constrained by inherent patient variability, strict ethical considerations, and stringent data privacy regulations. Furthermore, the lack of standardized wound imaging protocols across different healthcare facilities results in highly variable datasets, ultimately diminishing algorithmic precision and reliability. Critical issues surrounding patient safety, system dependability, algorithmic misclassification, and ultimate clinical accountability must be rigorously addressed before automated decision-making systems can achieve widespread clinical acceptance. Deploying these AI-assisted, hydrogel-based solutions in real-world clinical environments also presents logistical hurdles, as hospital infrastructures vary drastically in their current digital readiness.

To enable routine medical adoption, researchers and industry stakeholders must systematically overcome these core constraints. Looking forward, hydrogels may soon integrate flawlessly with ultra-stretchable electronics, microfluidic channels, and biofluid-powered energy harvesters, paving the way for entirely autonomous, self-regulating wound management platforms. Strategies such as the grafting of conductive polymers, graphene reinforcement, the 3D printing of hydrogel-embedded microchips, and the utilization of wireless transcutaneous power transfer are anticipated to significantly expedite the development of ultrathin, highly flexible, and skin-adaptive sensor–hydrogel systems. These next-generation hybrid materials possess the unprecedented capability to continuously monitor critical wound biomarkers—including pH, temperature, oxygenation, glucose, lactate, and bacterial load—relaying this real-time data to clinicians and patients via intuitive wearable interfaces and remotely accessible digital dashboards. This technological convergence holds immense potential to fundamentally transform chronic wound management, particularly for debilitating conditions such as diabetic foot ulcers, severe burns, and complex post-surgical wounds.

### 7.4. Concluding Remarks

Ultimately, future clinical frameworks may leverage predictive computational modeling, genetic profiling, and “digital twin” technologies to design truly patient-specific hydrogels, ushering in a new era of personalized wound care. The sustained development of smart hydrogel dressings must meticulously balance industrial scalability with cutting-edge sensor hybridization and AI-assisted autonomous management. Surmounting these multidisciplinary challenges will culminate in the realization of clinically intelligent systems capable of sensing, analyzing, dynamically adapting, and healing tissues with unprecedented precision.

## 8. Conclusions

Smart and bioengineered hydrogels have emerged as highly promising next-generation wound dressings, transcending the limitations of traditional materials by providing dynamic, adaptive, and multifunctional support for tissue repair. As comprehensively outlined in this review, smart hydrogels—particularly those exhibiting stimuli-responsive, injectable, self-healing, and 3D-printable properties—are capable of intelligently reacting to physiological fluctuations in pH, temperature, enzymatic activity, light exposure, and electrical signals within the localized wound microenvironment. This inherent adaptability facilitates the precise regulation of moisture levels, mechanical protection, and targeted therapeutic delivery, all of which are paramount for the effective management of complex and chronic wounds. Furthermore, functionalization significantly augments the therapeutic capabilities of these hydrogels through the strategic integration of antimicrobial agents, growth factors, active pharmaceuticals, living cells, conductive elements, and advanced nanocomposites. These sophisticated, multicomponent systems not only successfully inhibit microbial infection but also actively drive angiogenesis, rapid re-epithelialization, and comprehensive tissue regeneration. Rigorous physicochemical characterization, in vitro biocompatibility assessments, and in vivo validation models have robustly demonstrated their superior healing efficacy relative to conventional passive dressings. Clinical evaluation is either insufficient or has yet to be carried out. Furthermore, formidable challenges persist regarding large-scale manufacturing, stringent regulatory approval, long-term safety profiling, and overall cost-effectiveness. However, continuous advancements in material science, scalable fabrication technologies, and seamless integration with biosensors and AI-enabled monitoring systems are rapidly bridging this translational gap. Ultimately, intelligent and functionalized hydrogel platforms possess immense potential to fundamentally transform the landscape of wound care, paving the way for highly personalized, efficacious, and clinically translatable healing paradigms.

## Figures and Tables

**Table 1:** Overview of Smart Hydrogels in Wound Healing Models.

Hydrogel Matrix	Crosslinking Mechanism	Stimulus	Therapeutic Agent	In Vivo Model	Healing Outcome	Ref.
Double-network hydrogel	Schiff base dynamic bonding	pH	Resveratrol	Chronic wound animal model	Enhanced angiogenesis and collagen deposition	[80]
H-CuS@EGCG hydrogel	Injectable hydrogel composite	pH + NIR	EGCG + CuS nanoparticles	Infected rodent wound model	Nearly complete bacterial elimination and accelerated wound closure	[81]
CMCS/2-FPBA/TA-Fe hydrogel	Dynamic boronate ester + metal coordination	pH	Polyphenol complex	Diabetic wound animal model	ROS scavenging, macrophage M2 polarization	[82]
Nanozyme hydrogel	Enzyme-responsive network	pH + glucose	Glucose oxidase	Diabetic wounds	Improved angiogenesis and epithelial regeneration	[83]
PβAE/HA hydrogel	UV-crosslinked polymer network	pH	Bromelain enzyme	Animal wound model	Reduced inflammation and enhanced ECM remodeling	[84]
Carbon quantum dot nanofiber hydrogel	Electrospun composite	pH	CQDs	Chronic wound model	Real-time pH monitoring and wound healing support	[85]

## Data Availability

No new data were created or analyzed in this study. Data sharing is not applicable to this article.
